# The biocontrol effects and mechanisms of mycoparasitic *Alternaria alternata* against Chinese rose powdery mildew

**DOI:** 10.3389/fmicb.2025.1598458

**Published:** 2025-06-04

**Authors:** Yanping Tang, Ruotian Gao, Changle Ma, Jin Wang, Jing Li

**Affiliations:** ^1^College of Biological Science and Food Engineering, Southwest Forestry University, Kunming, Yunnan, China; ^2^Forest Resources Exploitation and Utilization Engineering Research Center for Grand Health of Yunnan Provincial Universities, Southwest Forestry University, Kunming, Yunnan, China; ^3^College of Landscape Architecture and Horticulture, Southwest Forestry University, Kunming, Yunnan, China; ^4^College of Forestry, Yunnan Forestry Technological College, Kunming, Yunnan, China

**Keywords:** Chinese rose powdery mildew, biological control, Mycoparasite, *Podosphaera pannosa*, Chitinase

## Abstract

**Introduction:**

Chinese rose powdery mildew, caused by *Podosphaera pannosa*, is a devastating disease which has a significant impact on plants' ornamental and economic value. Strain KMR13, which exhibited pronounced mycoparasitic activity against *P. pannosa*, was isolated during the initial phase of this study; however, the underlying mechanism remains to be elucidated.

**Methods:**

In order to analyze the biological control and mycoparasitism mechanisms, the present study was carried out to sequence the whole genome of strain KMR13 using a combination of second-generation Illumina and third-generation nanopore platforms, to mine chitinase genes from the KMR13 genome, and to screen for chitinase genes related to mycoparasitism by detecting the expression of the genes at different time periods of sporulation induction.

**Results:**

The results revealed a genome size of 33,532,117 base pairs (bp) with a GC content of 50.97%, encoding 12,545 genes and 379 non-coding RNAs. Functional annotations using NR, GO, KOG, Pfam, and KEGG databases identified 12,355, 8,208, 1,871, 7,911, and 7,657 genes, respectively. A total of 15 GH18 family genes were mined in KMR13, and a total of 10 chitinase genes were detected to be expressed in the transcriptome under spore induction, 5 genes were consistently up-regulated for expression after induction, and 5 genes had the highest expression at 24h of induction. RT-qPCR analysis of 5 genes with high expression as well as high fold expression showed significant differential expression for all genes, with the highest expression at 24 h of induction. Up-regulated expression of *KMRChis* after induction is likely to play a role in disrupting the spore wall during mycoparasitic process of strain KMR13. Field trials demonstrated that KMR13 conidial suspensions significantly suppressed *P. pannosa*-induced powdery mildew, although the control efficacy was lower than that observed under greenhouse conditions.

**Discussion:**

These findings collectively highlight the potential of KMR13 as a biological control agent and provide a theoretical foundation for eco-friendly management of Chinese rose powdery mildew.

## 1 Introduction

The Chinese rose, belonging to the genus *Rosa* in the family Rosaceae, is one of the most popular species in the world due to its ornamental and economic value (Feng et al., 2015; Qi et al., 2018). Known as the queen of flowers (Yu et al., 2023) the Chinese rose is an economically and culturally important species; it also has historical significance, appearing as a decoration on 5,000-year-old Asian pottery (Wang, 2007). The Chinese rose is of greater economic importance than any other flowering plant, and it is grown and sold worldwide as a garden plant, in pots, or as cut flowers, with the latter accounting for approximately 30% of the flower market (Nybom and Werlemark, 2017). As a popular ornamental plant, the Chinese rose faces many pathogens during the planting process, with *Podosphaera pannosa*, an obligate living mycoparasitic fungus, being a particularly harmful example (Cook et al., 1997). The Chinese rose powdery mildew caused by this pathogen is as a major disease in the global cut rose industry. All over the globe, Chinese roses, whether cultivated in the open air or in greenhouses, are susceptible to *P. pannosa*, which seriously threatens production and the conservation of resources. Chinese rose powdery mildew affects the shoots, leaves, and flower buds, and most of the young leaves are left in a rolled-up state post-infection. The infection site produces a large amount of white powder, and in serious cases, the plant's growth and flowering are affected, affecting the ornamental effect. Therefore, in-depth research on Chinese rose powdery mildew and its pathogens, as well as exploring effective control measures, is of great significance to the healthy development of the Chinese rose industry.

At present, powdery mildew is generally controlled by planting resistant varieties and applying chemical fungicides (Kiss et al., 2004). However, over time, powdery mildew has become less sensitive to chemical compounds, driving the development of fungicide resistance in the pathogen population (Vielba-Fernández et al., 2020). Excessive use of chemical fungicides is also harmful to the environment and human and animal health. The use of resistant cultivars is limited. Therefore, it is necessary to find feasible and effective methods to control Chinese rose powdery mildew. As science and technology have progressed, the importance of biological control has become clear. The biological control method has obvious advantages in terms of safety, environmental friendliness, and high efficiency, so it is regarded as an ideal replacement for chemical pesticides which will help promote the sustainable development of agricultural production. Biological control agents (BCAs) can provide alternative methods for preventing or inhibiting powdery mildew for some crops (Kiss, 2003). It is reported that several commercial biological control agents based on *Trichoderma harzianum, Aureobasidium pullulans, Bacillus subtilis, Streptomyces griseoviridis*, and *Gliocladium virens* can be used to fight against different plant fungal pathogens (Spadaro and Droby, 2016; Liu et al., 2013). Biological bactericidal products such as *Ampelomyces quisqualis* (AQ10) biological bactericide, Q-fect, Powdercare^®^ and Sporodex^®^ have been registered and listed in some countries/regions (Park et al., 2010). In their research, Xie et al. (2021) observed that a concentration of 4 × 10^5^ CFU/ml of *B. subtilis* exhibited a significant effect on wheat powdery mildew caused by *Blumeria graminis f. sp. tritici*, inhibiting the germination of conidial germ tubes and the normal development of appressoria. Newman et al. (1999) demonstrated that the combined application of Quaternary benzophenanthridine alkaloids (QBAs) and piperalin significantly enhanced control efficacy against rose powdery mildew caused by *Sphaerotheca pannosa var. rosae*. Sawant et al. (2017) demonstrated that combining *Trichoderma afroharzianum* strain NAIMCC-F-01938 with safe fungicides significantly enhanced its efficacy against grapevine powdery mildew caused by *Erysiphe necator Schw*.

The biological control mechanisms were comprised of competition, antagonism, mycoparasitism, inducing plant to generate resistance and promoting growth of plant. Mycoparasitism is an important mechanism by which biocontrol fungi antagonize plant pathogens (Sun et al., 2015). At present, the research on the mechanism of action of mycoparasitism mainly includes two aspects of enzyme and toxin, enzyme refers to mycoparasitic fungus can produce enzymes that degrade the cell wall of the host fungus, toxin is mycoparasitic fungus interact with host fungus to produce antimicrobial secondary metabolites, which are able to kill the host fungus, toxin after the action of the growth of the pathogenic fungus will be inhibited, but the cell wall will not be destroyed, but the cell will appear to be deformed, and the contents will be condensed and overflowed to make the cell death (Deacon, 2005). Additionally, mycoparasites secrete cell-wall-degrading enzymes, including chitinase and glucanase (Almeida et al., 2007), during the process of mycoparasitic activity. They also produce various secondary metabolites (Khan et al., 2020), such as polyketides (Kong et al., 2021; Zhao et al., 2020), melanin, citric acid, and 3-nitropropropionic acid (Li et al., 2022). Of these, chitinase and β-1,3-glucanase play a crucial role in the breakdown of mycoparasite cell walls, and toxins such as secondary metabolites can also kill the host fungus; the mycoparasitic fungus can then absorb host nutrients. Chitinase, as an important protein in the interaction between pathogens and host plants, has become a hot topic in the study of plant resistance to pests and diseases (Malik, 2019). Chitin, an important component of fungal cell walls, is a multimer consisting of N-acetylglucosamine units [N-acety1-D-(+)-glucosamine] linked by β-1,4 glycosidic bonds (Hossin et al., 2021). Fungi are the major group of chitinase producers among microorganisms. In fungi, chitinases are involved in morphogenesis, cytokinesis, autolysis, nutrient uptake and parasitism, and are also an important mode of biocontrol (Duo-Chuan, 2006). It has been demonstrated that mycoparasitic *Trichoderma* inhibit phytopathogenic fungi by secreting cell wall degrading enzymes, such as chitinase, and secondary metabolites (Mohammad Hood et al., 2023).

Thus far, mycoparasitic *A. alternata* has not been used to treat *P. pannosa*-induced Chinese rose powdery mildew. This study marks the first attempt to use this strain to control this disease. A strain KMR13 with significant mycoparasitic effect on *P. pannosa* was isolated in the early part of this study, but its mechanism of action is not clear (Tang et al., 2025). Therefore, whole genome sequencing of strain KMR13 using a combination of second-generation Illumina and third-generation nanopore platforms, mining of chitinase genes in strain KMR13, and screening of mycoparasite associated chitinase genes by combining the transcriptome and RT-qPCR data will help to better understand the mechanism of this disease and provide a theoretical basis for a deeper understanding of the biocontrol mechanism and efficient development and utilization of strain KMR13.

## 2 Materials and methods

### 2.1 Test material

#### 2.1.1 Pathogen

In 2023, *P. pannosa* was isolated from diseased Chinese rose (Rosa chinensis) leaves exhibiting typical powdery mildew symptoms (characterized by white powdery mildew on the adaxial surface) collected from greenhouse-grown plants in the suburban region of Kunming City, Yunnan Province, China (24°23′N, 102°10′E).

#### 2.1.2 Test strains

The mycoparasitic strain KMR13 was isolated and screened from *P. pannosa* in our laboratory. It was identified as *Alternaria* sp. based on morphological characteristics and ITS sequence analysis (Tang et al., 2025), and is currently deposited in the Department of Biochemistry, College of Biological and Food Engineering, Southwest Forestry University, Kunming, China (Accession No. SWFU-BC-2023-KMR13). Strain KMR13 was cultured on PDA at room temperature for 5 days. Sufficient mycelium was collected for total genomic DNA extraction.

#### 2.1.3 Test medium

We used two media: Potato Dextrose Agar (PDA): (containing :200 g of potato, 20 g of glucose, 20 g of agar, 1,000 mL of sterile water, adjusted to natural pH), and Potato Dextrose Broth (PDB): (containing: 200 g of potato, 20 g of glucose, and 1,000 mL of sterile water, adjusted to natural pH).

### 2.2 Mycoparasitism assay

We began with high-temperature sterilization of a Petri dish containing filter paper. As mall amount of sterile water was dripped onto the filter paper in a sterile environment, then the petiole was wrapped in cotton and sprayed with water to maintain the humidity. A small pile of *P. pannosa* (a causal agent of Chinese rose powdery mildew) spore mass was shaken onto the filter paper, and the test strains were inoculated with a diameter of about 5 mm on the spore mass. The control sample was one which was not exposed to *P. pannosa*. Each treatment was repeated 3 times and cultivated at 28°C. Observations were carried out every 2 days and continuously recorded for 5 days.

### 2.3 Biosafety assessment

Strain KMR13 was streaked on PDA and incubated at 28°C for 3 days. A single colony was aseptically transferred using an inoculation loop into PDB and cultured at 28°C with shaking (160 rpm) for 48 h. The resulting fungal culture was then adjusted to a conidial suspension concentration of 1 × 10^6^ CFU/mL using sterile distilled water. The fungal conidial suspension with a concentration of 1 × 10^6^ CFU/mL was evenly sprayed onto the seedling leaves using a handheld sprayer. Seedlings inoculated with sterile water were used as the control. Each treatment was repeated 3 times, then covered with plastic film for moisture retention. The disease symptoms were observed daily. On the 7th day, the incidence rate, disease index, and safety level were assessed. The disease grading standards are the same as those in Li et al. (2014), with slight modifications. Level 1: asymptomatic, with no disease spots on the plant leaves; Level 2: mild reaction, with scattered disease spots distributed on the plant leaves; Level 3: moderately susceptible, where 1/4 to 1/2 of the leaves of the plant are dead and growth is inhibited; Level 4: seriously susceptible, where 1/2 to 3/4 of the leaves of the plant are dead and growth is severely inhibited. When 0 ≤ disease index <5, the safety level is no symptom (NS); when 5 ≤ disease index <10, the safety level is lightly susceptible (LS); when 10 ≤ disease index <50, the safety level is moderately susceptible (MS); when disease index > 50, the safety level is severely susceptible (SS).


$$\begin{array}{l} Incidence\;rate\left(\%\right)=\frac{number\;of\;plants\;affected}{total\;number\;of\;plants\;surveyed}\times 100\;\\ Disease\;index\left(\%\right)\\ =\frac{\sum \left(number\;of\;disease-grade\;plants\;\times \;number\;of\;corresponding\;plant\right)}{total\;number\;of\;plants\;surveyed\;\times \;highest\;grade\;index}\times 100\; \end{array}$$

### 2.4 Control effect of biocontrol fungus on Chinese rose powdery mildew

#### 2.4.1 Control effect of indoor potted plants

Indoors at 15–20°C, relative humidity 60–65%, biocontrol fungus (1 × 10^6^ cfu/mL) was distributed evenly on the upper and lower surfaces of diseased Chinese rose leaves via spraying until the conidial suspension dripped onto the leaves. Aseptic water treatment was used as a control. Each treatment was repeated 3 times, for 5 potted plants. On the 7th day, the control effect of biocontrol fungus was investigated, and the disease index and control effect were calculated. Disease grading was conducted according to Huang (2017). Level 0: no disease or nearly no disease; Level 1: scattered spots on the leaves; Level 2: more than 1/4 of the leaves show lesions, and plant growth is suppressed; Level 3: more than 2/3 of the leaves show lesions, and plant growth is severely suppressed; Level 4: more than 3/4 of the leaves show lesions, and the plant is nearly or completely dead.


$$\begin{array}{l} Control\;effect\left(\%\right)\\ =\frac{control\;average\;disease\;index\;-\;treatment\;average\;disease\;index}{control\;average\;disease\;index}\times 100\; \end{array}$$

#### 2.4.2 Field control effect

Field greenhouse control effect tests were conducted at Yunnan Baihu Horticultural Science and Technology Co., Ltd. at daytime temperatures of 23–28°C and relative humidity of 55%−65%, and at night temperatures of 10–15°C and relative humidity of 85%−95%, biocontrol fungus (1 × 10^6^ cfu/mL) was distributed evenly on the upper and lower surfaces of diseased Chinese rose leaves via spraying until the conidial suspension dripped onto the leaves. Aseptic water treatment was used as a control. Each treatment was applied to 10 Chinese roses twice at intervals of 7 days and the treatment was repeated thrice. The control effect of biocontrol fungus was investigated on the 14th day after application, and the disease index and control effect were calculated.

### 2.5 Genome assembly and evaluation

Strain KMR13 was cultured on PDA at room temperature for 5 days, and then the mycelium was scraped off with a scalpel and snap-frozen in liquid nitrogen and sent to Wuhan Benetech for whole genome sequencing. The initial genome assembly was conducted using the NECAT software (https://github.com/xiaochuanle/NECAT). Two subsequent rounds of three-generation error correction were performed on the initial assembly results using three-generation ONT sequencing data; this was achieved using Racon software (version: 1.4.11). After that, two rounds of second-generation error correction were conducted on the initial assembly results after three-generation error correction using second-generation sequencing data; this was achieved using Pilon software (version: 1.23). In the case of low heterozygosity species, the corrected genome represents the final assembly result. In the case of highly heterozygous species, the final assembly (Draft Genome) is obtained by de-hybridizing the corrected genome using the software Purge_hap lotigs (version 1.0.4).

### 2.6 Gene structure prediction

BRAKER software (version:2.1.4) is mainly used for gene prediction. First, GeneMark-EX is used to train the model, and then AUGUSTUS is utilized for prediction. RepeatMasker (version: open-4.0.9) is used to make repeated comments based on RepBase library (http://www.girinst.org/repbase). Then, RepeatModeler (version: open-1.0.11) is used to build a database for denovo prediction based on its own sequence characteristics, and RepeatMasker (version: open-4.0.9) also used to compare and predict repeated sequences. Finally, all the repeated prediction results are combined to eliminate redundancy, and the final genome repeated sequence set Combined TEs is obtained. Based on the structural characteristics of tRNA, tRNAscan-se (version: 1.23) was used to find the tRNA sequence in the genome. The rRNA database was used to predict rRNA, and INFERNAL (version: 1.1.2) based on the Rfam database was used to find ncRNA sequences in the genome, such as snRNA, miRNA, etc.

### 2.7 Gene function annotation

Functional annotation of genes involves the classification of gene functions and metabolic pathways based on existing databases. This involves the prediction of motifs, structural domains, protein functions, and the metabolic pathways in which they are located. To obtain comprehensive gene function information, we performed gene function annotations of nine databases, including Nr, Pfam, KOG, Uniprot, KEGG, GO, Pathway, Refseq, and Interproscan.

### 2.8 Carbohydrate enzyme annotation

The Carbohydrate-Active enZYmes Database (CAZy) focuses on analyzing genomic, structural, and biochemical information of carbohydrate-active enzymes. It comprises six main categories: glycoside hydrolases (GHs), glycosyltransferases (GTs), polysaccharide lyases (PLs), auxiliary activities (AAs), carbohydrate esterases (CEs), and non-catalytic carbohydrate-binding modules (CBMs). Protein sequences were annotated using HMMER (version: 3.2.1) based on the CAZy database, with filtering parameters set at E-value < 1e-18 and coverage > 0.35.

### 2.9 Chitinase gene mining and induced expression

#### 2.9.1 Chitinase gene mining

Members of the *GH18* gene family were screened based on the results of annotation of available genomic information. The *GH18* gene family members were obtained from the pfam (http://pfam.xfam.org/) database with sequence number PF00704 (El-Gebali et al., 2019), and this was used to screen the strain KMR13 genome to identify GH18 family candidate genes. The online tools Conserved Domain Database (CDD) (Available online: http://www.ncbi.nlm.nih.gov/cdd/) (Lu et al., 2020) and SMART (Available online: http://smart.embl-heidelberg.de/) (Letunic and Bork, 2018) were used to validate the above candidate genes, screen the sequences containing the conserved structural domains of the *GH18* gene family, delete the duplicates and deletion of structural domains, and then take the intersection of the two results to obtain the candidate genes of the *GH18* gene family.

#### 2.9.2 Induced expression of chitinase genes

After the collection of powdery mildew spores, the powdery mildew spores were repeatedly frozen and thawed at −20°C, and then ground with a mortar and pestle, and the ground powdery mildew spores were collected and observed under a microscope, and then the powdery mildew spores were sterilized under the condition of 121°C for 20 min, which were then used as powdery mildew spore walls of the inducers. After activation of strain KMR13 on PDA, the second-generation cultured mycelium was inoculated into 100 ml of PDB and incubated in a constant temperature shaker at 28°C, 150 r/min for 24 h. The spores were then induced with 2.5 g/L spores for 0, 24, 48, 72, and 96 h. There were three biological replicates for each group of treatments. After the induction was completed, the induced mycelium was taken out under aseptic conditions, frozen in liquid nitrogen and stored, and then submitted to Hangzhou Lianchuan Biotechnology Co. for transcriptome sequencing. The expression of the *GH18* gene family was screened for experimental and control groups, then analyzed and heatmapped using TBtools.

#### 2.9.3 RNA extraction and RT-qPCR

RNA was extracted using the Tengen RNA Extraction Kit, and the first strand of cDNA was synthesized using the Reverse Transcription Kit, using a 20 uL reaction system. The target genes with high expression or large fold difference in expression were screened for quantitative real-time polymerase chain reaction (RT-qPCR). cDNA was used as the template and actin as the internal reference gene, and primers were designed with Primer Primer5 software, and the sequences of the internal reference and gene primers are shown in Table 1. Relative quantification was calculated using the 2-ΔΔCt, and the data were analyzed by one-way ANOVA using SPSS 26.0 software.

**Table 1:** RT-qPCR primers.

**Gene name**	**Forward primer**	**Reverse primer**
*KMRChi7*	TCGCCAGCTTGATACACACC	ACCTCTCACGCTCTCATTGT
*KMRChi9*	CTTACGCTCCCGCTCCTTAC	GTGAACTCCGCTGGCATAGA
*KMRChi10*	GGGAATTGGATTGGTTCTTCCC	AACCAGTCTCACTCTAGCCC
*KMRChi12*	GTCACATACGATACCCCGCA	ATCCTTCTGAGTGAGCGAGC
*KMRChi15*	CGCCCAATACGACTTCTTTCG	CTCGCCTTATTCCTGTGTTGC

## 3 Results

### 3.1 Mycoparasitism assay

**Figure 1:**
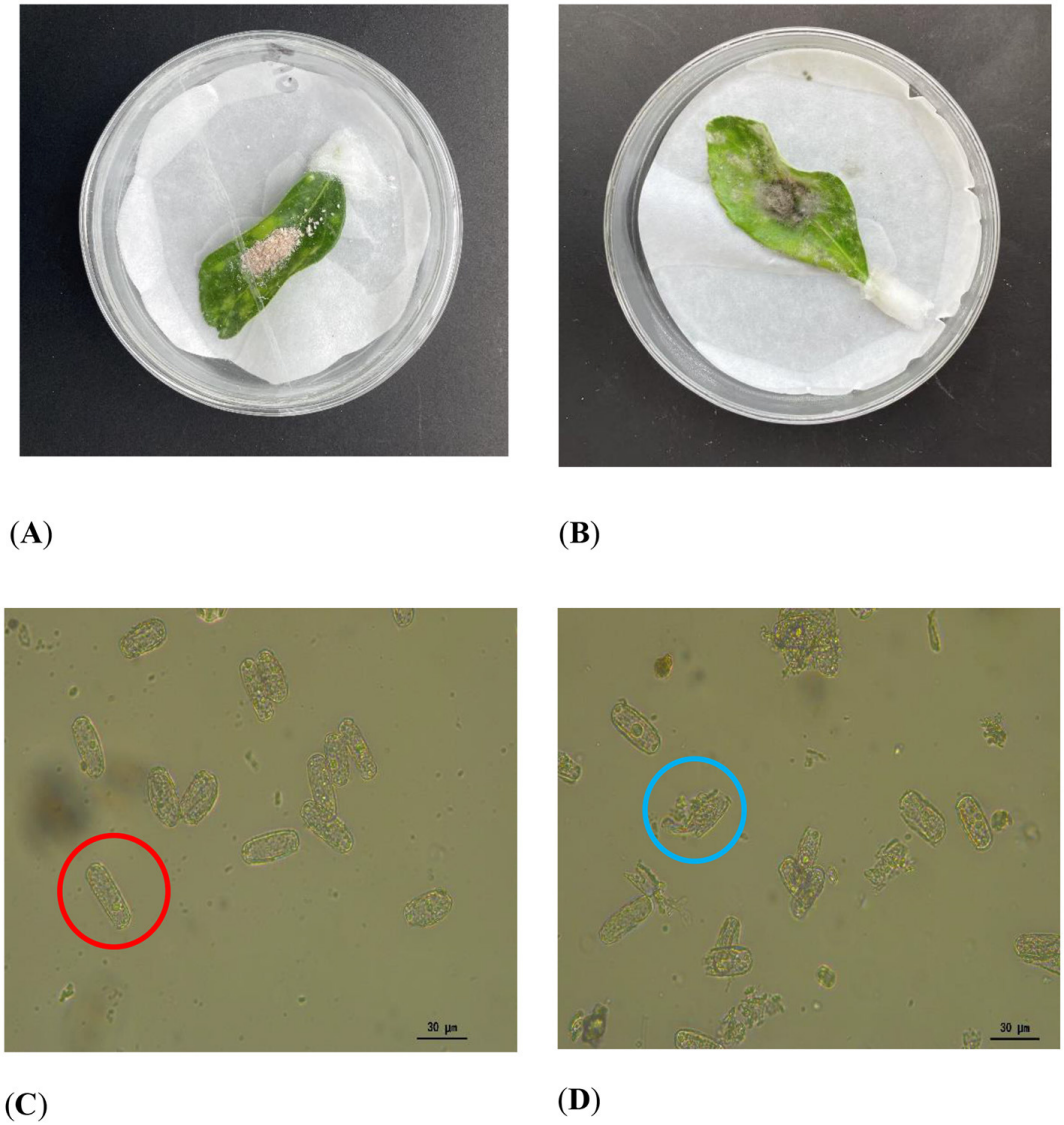
Mycoparasitism assay: **(A, B)** Colonization of *P. pannosa* (a causal agent of Chinese rose powdery mildew) spore masses by strain KMR13. **(C, D)** Microscopic characterization of spores after treatment of the strain. **(A, C)** Control, intact spores are circled in red; **(B, D)**: treatment, blue circles indicate rupture of the spore wall and spillage of the contents.

Mycoparasitism assays revealed that the strain KMR13 could grow on the *P. pannosa* spore mass and cover it, as shown in Figures 1A, B. Microscopic examination of the *P. pannosa* spore mass covered by the hyphae revealed disruption to the cell walls, inducing leakage of intracellular contents. In contrast, the control group with the *P. pannosa* spore mass that was not exposed to the tested strain showed no evidence of broken cell walls, as illustrated in Figures 1C, D.

### 3.2 Biosafety assessment

**Figure 2:**
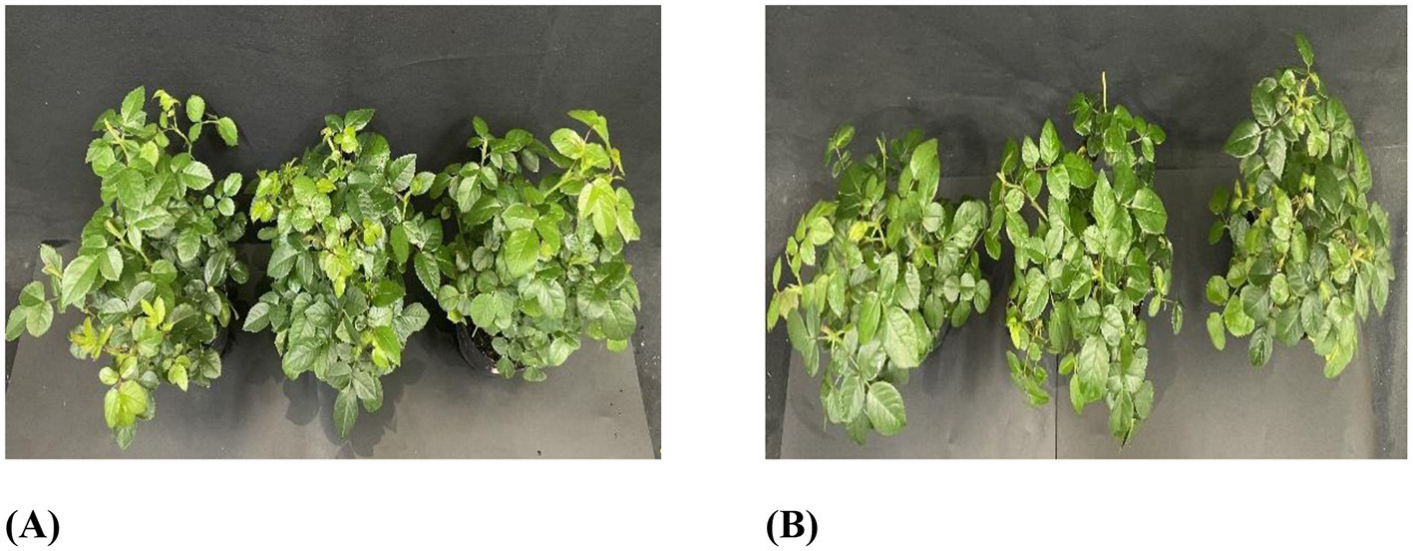
The safety of strain KMR13 on Chinese roses: **(A)**: Control; **(B)**: treatment.

The safety assessment revealed that after the KMR13 fungal conidial suspension was applied for 7 days, there were no changes in growth and leaf color compared to the control group. The Chinese rose leaves showed no disease spots, and both the incidence rate and disease index were 0, resulting in a safety level of NS. The results are shown in Figure 2.

### 3.3 Control effect of biocontrol fungi on Chinese rose powdery mildew

#### 3.3.1 Control effect of indoor potted plants

**Figure 3:**
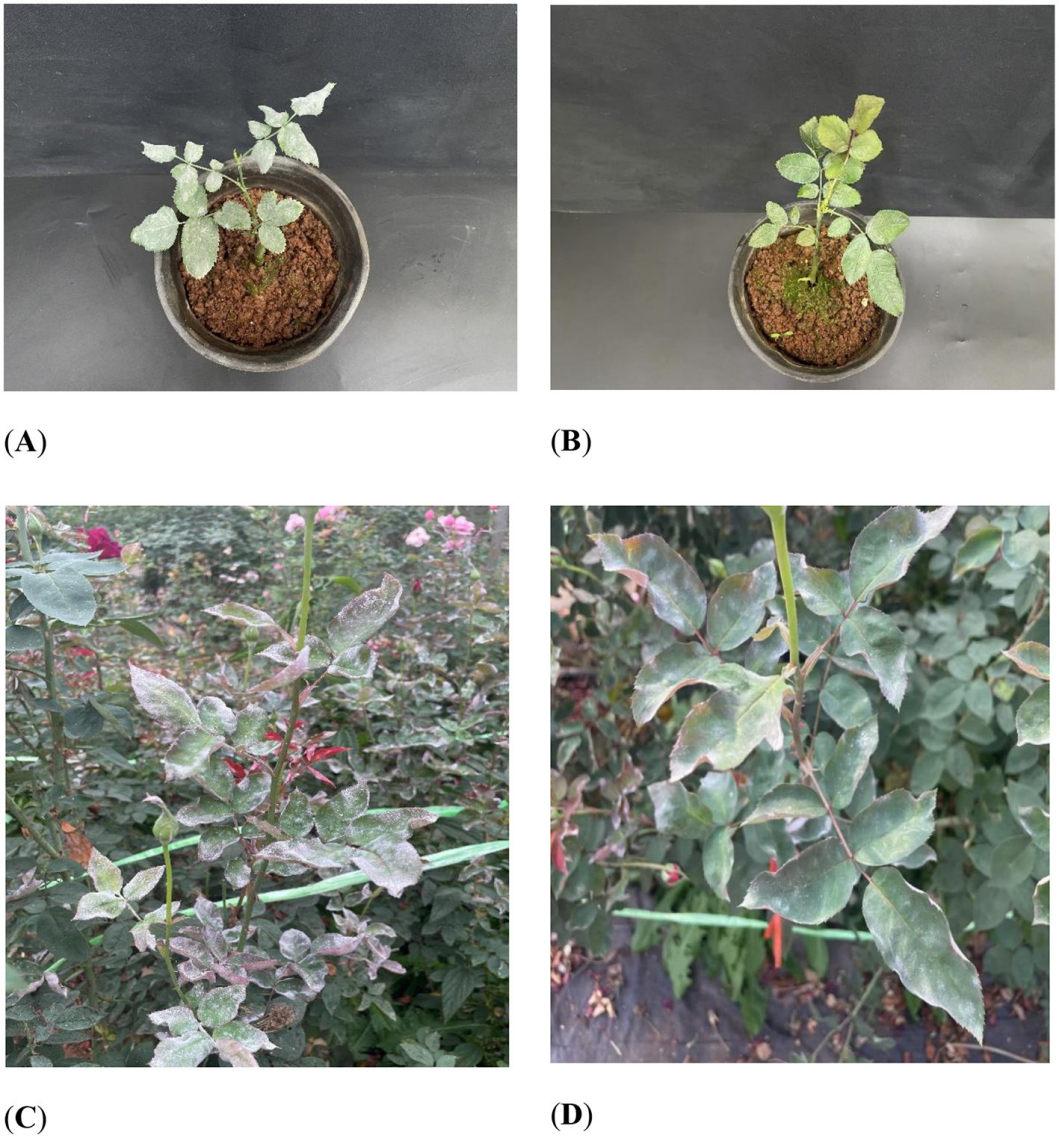
Control effect of biocontrol fungi on Chinese rose powdery mildew: **(A B)** control effect of biocontrol fungi on Chinese rose powdery mildew in indoor potted plants; **(C, D)** control effect of biocontrol fungi on Chinese rose powdery mildew in fields. **(A, C)** Control; **(B, D)** treatment.

The effect of KMR13 fungal conidial suspension on Chinese rose powdery mildew is shown in Figures 3A, B. The disease index of Chinese rose powdery mildew after treatment with KMR13 fungal conidial suspension was 32.78, while the disease index of the control group was 88.33, and the disease index of the KMR13 fungal conidial suspension was significantly lower than that of the control. The control effect of KMR13 fungal conidial suspension on Chinese rose powdery mildew was 62.89%, which indicated that the treatment of KMR13 fungal conidial suspension was successful at controlling Chinese rose powdery mildew.

#### 3.3.2 Field control effect

The field control effect of KMR13 fungal conidial suspension on Chinese rose powdery mildew was investigated, and the results are shown in Figures 3C, D. The disease index of KMR13 fungal conidial suspension was 41.11 in the treated group, while that of the control group was 92.22, and the control effect of KMR13 on Chinese rose powdery mildew was 55.42%. The results showed that KMR13 fungal conidial suspension had a significant inhibitory effect on Chinese rose powdery mildew, although the control effect in a field environment was lower than that in the greenhouse. This proved that KMR13 is a biocontrol fungus that can be used to control Chinese rose powdery mildew.

### 3.4 Genome assembly and evaluation

The raw data from the second-generation sequencing, were filtered and assembled. Eleven coatings were obtained from the assembled KMR13 genome, with a total genome size of 33.53Mb and a GC base content of 50.97%, of which the longest sequence length was 6832736bp and N50 was 3084465bp long. See Table 2 for details.

**Table 2:** Essential features of the genome of strain KMR13.

**Item**	**Value**
Total length (bp)	33,532,117.00
Total number	11.00
GC content (%)	50.97
N50 (bp)	3,084,465.00
N90 (bp)	2,443,235.00
Average (bp)	3,048,374.27
Median (bp)	2,580,072.00
Min (bp)	50,609.00
Max (bp)	6,832,736.00

The genome assembly results were evaluated for continuity, consistency, and completeness. The N50 of strain KMR13 was 3084465 bp, indicating a high level of continuity. The second-generation data-matching rate was 98.65%, and the coverage rate was 100.00%. The second-generation comparison rate and coverage—generally exceeded 90%, exhibiting better genome consistency. In BUSCO evaluation, the proportion of Complete BUSCOs (C) generally surpasses 90%, and the genome integrity is good. See Table 3 for details.

**Table 3:** BUSCO evaluation statistics.

**Item**	**Number**	**Percent (%)**
Complete BUSCOs (C)	756	99.7
Complete and single-copy BUSCOs (S)	755	99.6
Complete and duplicated BUSCOs (D)	1	0.1
Fragmented BUSCOs (F)	1	0.1
Missing BUSCOs (M)	1	0.2
Total BUSCO groups searched	758	100.0

### 3.5 Gene structure prediction

After using the Gene Mark-EX training model, AUGUSTUS was called to predict the pre-obtained coding genes. The total number of predicted genes was 12,545, the average mRNA length was 2,393.83, and the average length of CDS was 1,398.91. See Table 4 for details.

**Table 4:** Statistics of coding gene prediction results.

**Item**	**Number**
The total number of genes	12,545
The average of mRNA length (bp)	2,393.83
The average CDS length of per gene (bp)	1,398.91
The average number of exons per gene	2.8
The average exon length (bp)	792.93
The average intron length (bp)	98.36
The total number of exons	35,078
The total number of introns	22,533
The total intron length (bp)	2,216,316

**Figure 4:**
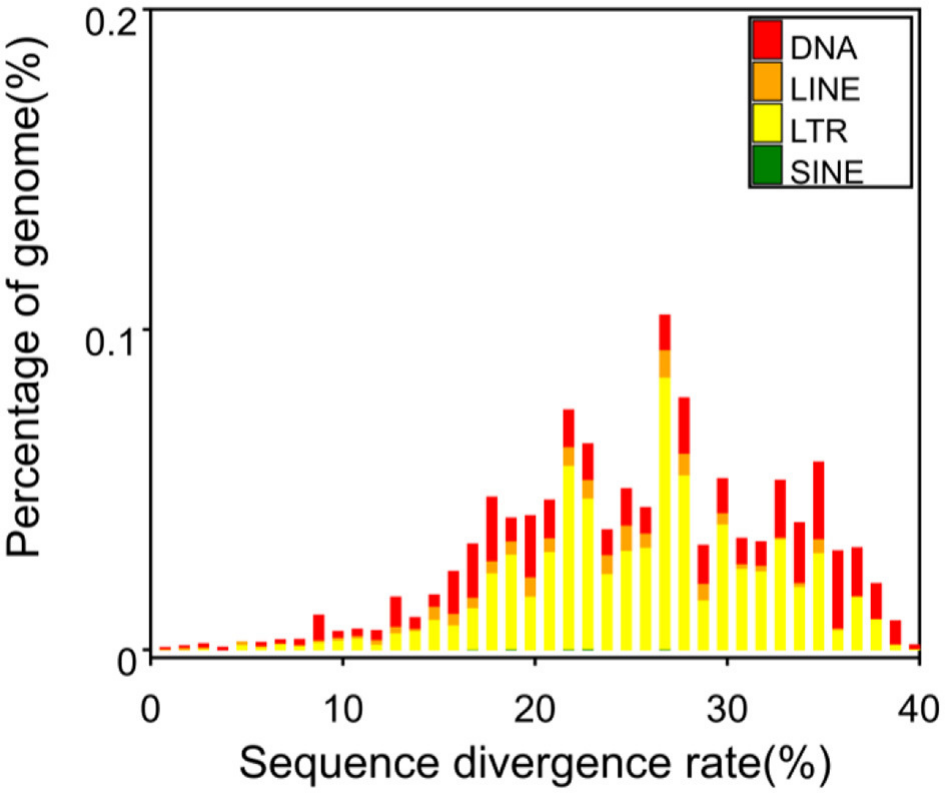
Percentage of repeated sequences of various types. DNA, DNA transposon; LINE, long interspersed repeat sequence (not LTR). One of non-LTR was recorded: a kind of reverse transcription transposon, including L1, R2, Jockey, etc. LTR (long terminal repeat): long terminal repeat sequence. SINE: short interspersed repeat sequence. One non-LTR was recorded: a kind of reverse transcription transposon, such as V-SINE, AmnSINE, CORE-SINE, etc.

Figure 4 illustrates the predictions generated by RepeatModeler software, which indicate that DNA constitutes 0.69% of the genome, long scattered repeat sequences account for 0.30% of the genome, there are no short scattered repeat sequences, and long terminal repeat sequences represent 1.15% of the genome. A total of 379 non-coding RNAs were identified in this genome, comprising 64 rRNAs, 122 tRNAs, and other non-coding RNAs.

### 3.6 Gene function annotation

According to the total annotation of genome data, 12,545 genes were predicted, of which 12,387 genes (98.74%) had at least one annotation (compared to a database). The Nr database contained the greatest number of annotated genes (12,355, accounting for 98.49% of the total number of genes). The specific annotation results of different databases are shown in Table 5, and annotation results from subsequent research are provided to determine the target functional genes.

**Table 5:** Functional annotation results of coding genes.

**Item**	**Count**	**Percent (%)**
All	12,545	100.00
Annotation	12,387	98.74
KEGG	7,657	61.04
Pathway	3,599	28.69
Nr	12,355	98.49
Uniprot	12,294	98.00
GO	8,208	65.43
KOG	1,871	14.91
Pfam	7,911	63.06
Interproscan	11,000	87.68
Refseq	12,297	98.02
Tigerfam	2,668	21.27

#### 3.6.1 Nr annotation results

**Figure 5:**
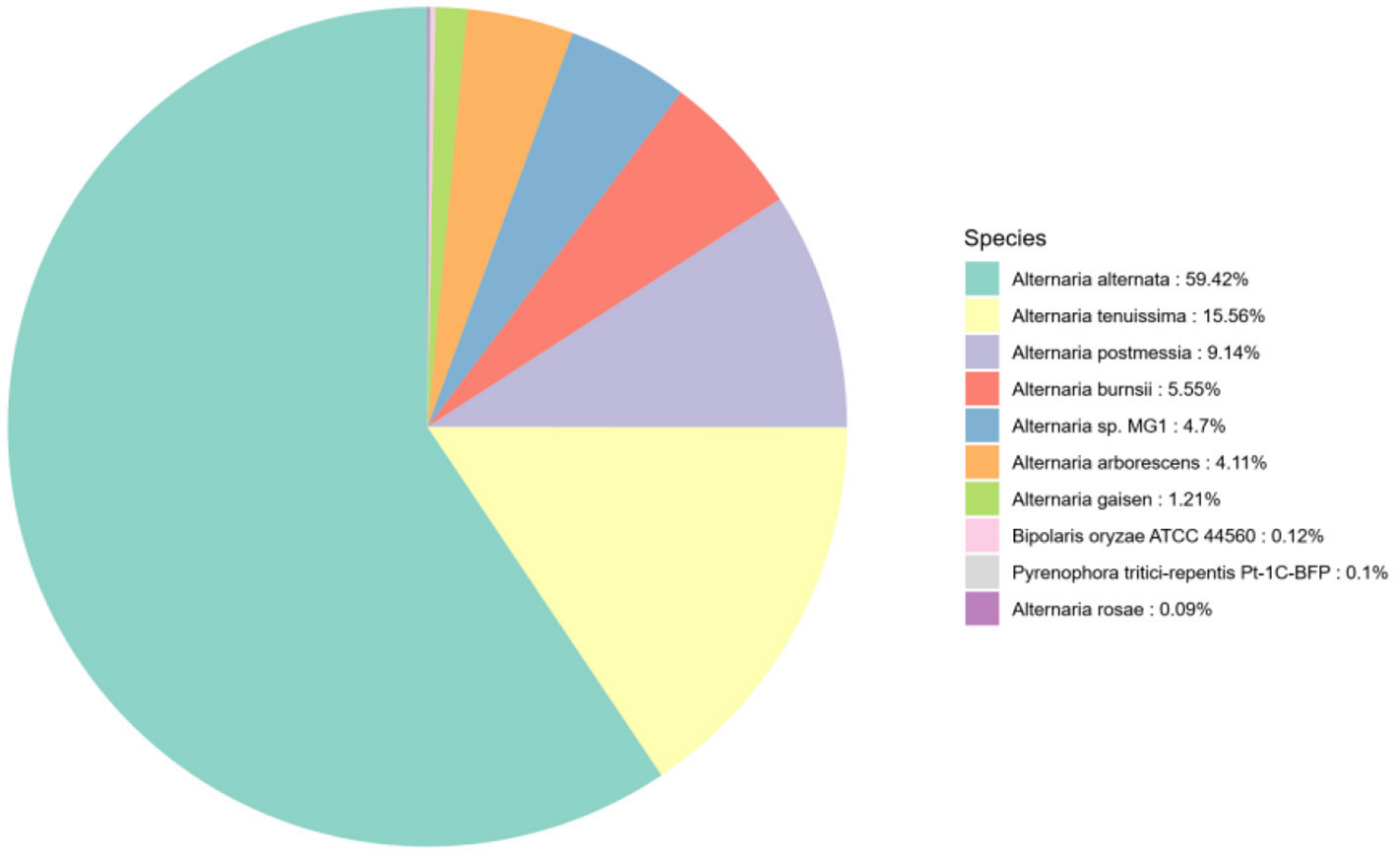
Species distribution map of sequences aligned to the Nr database. Different colors represent different species.

The results of the annotation are compared with the Nr database, and the top 10 species with the most comparisons are classified into other categories. Of the 12,355 annotated genes in the Nr database, *A. alternata* accounts for the highest proportion, 59.42%. The species distribution map is shown in Figure 5.

#### 3.6.2 GO Classifieds

**Figure 6:**
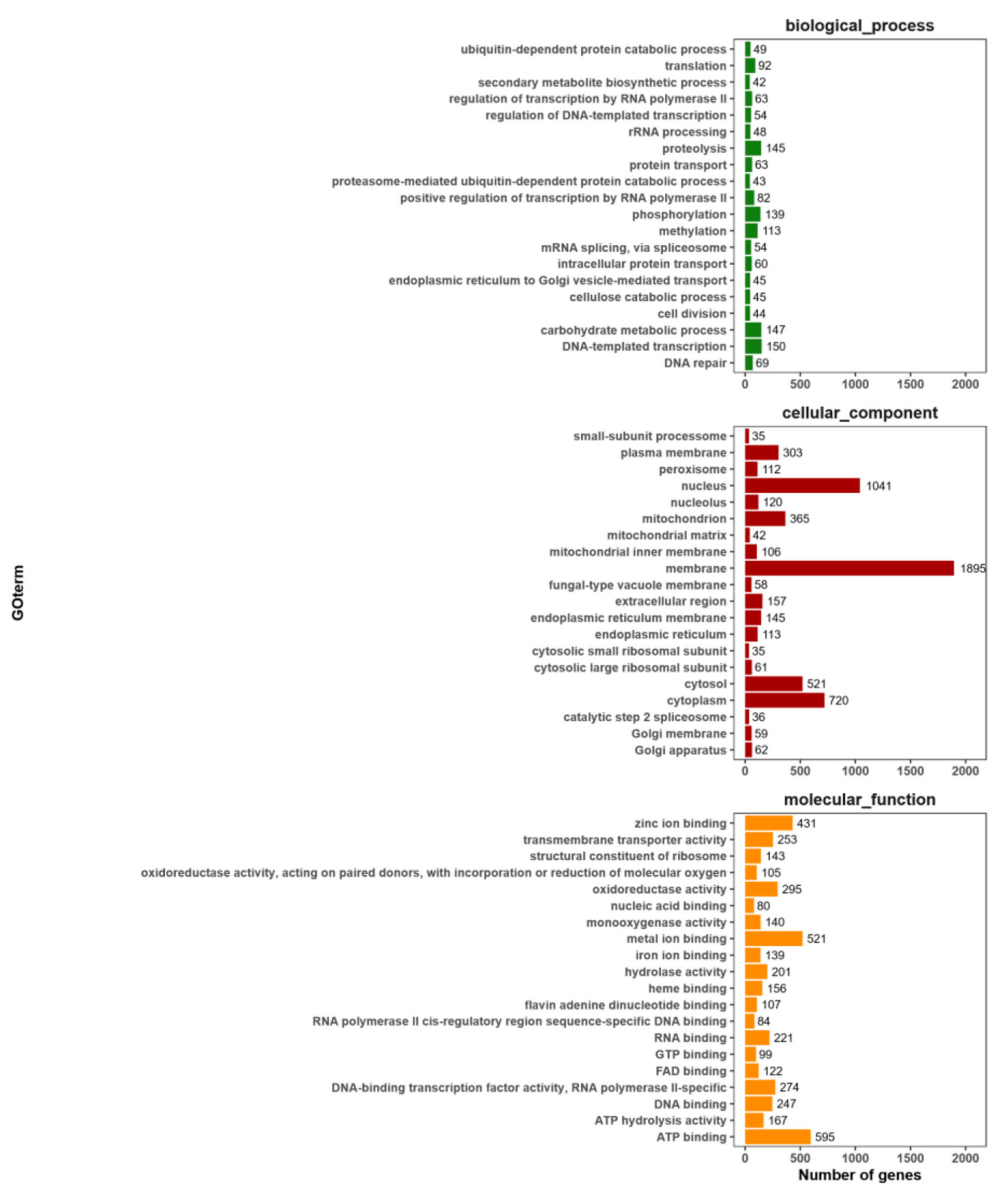
Statistical chart of GO function annotation classification. The horizontal axis represents the various categories of GO, while the vertical axis represents the number of genes. This figure shows the gene enrichment of each secondary function of GO against the background of all genes, highlighting the significance of each secondary function in this context.

The GO annotation information was extracted, and the top 20 secondary classification items with the most annotations were selected. A total of 8,208 annotated genes were identified, representing 65.43% of the total number of genes. The results of the gene function analysis, conducted from three perspectives—the cellular component, molecular function, and biological processes are presented in Figure 6. The protein encoded by KMR13 is predominantly associated with a range of biological processes, including DNA-templated transcription, the carbohydrate metabolic process, protein hydrolysis, and phosphorylation. The gene is found to be enriched in cell components, including the membrane, nucleus, and cytoplasm, as well as in molecular functions such as ATP binding, metal ion binding, zinc ion binding, and oxidoreductase activity.

#### 3.6.3 KOG classification

**Figure 7:**
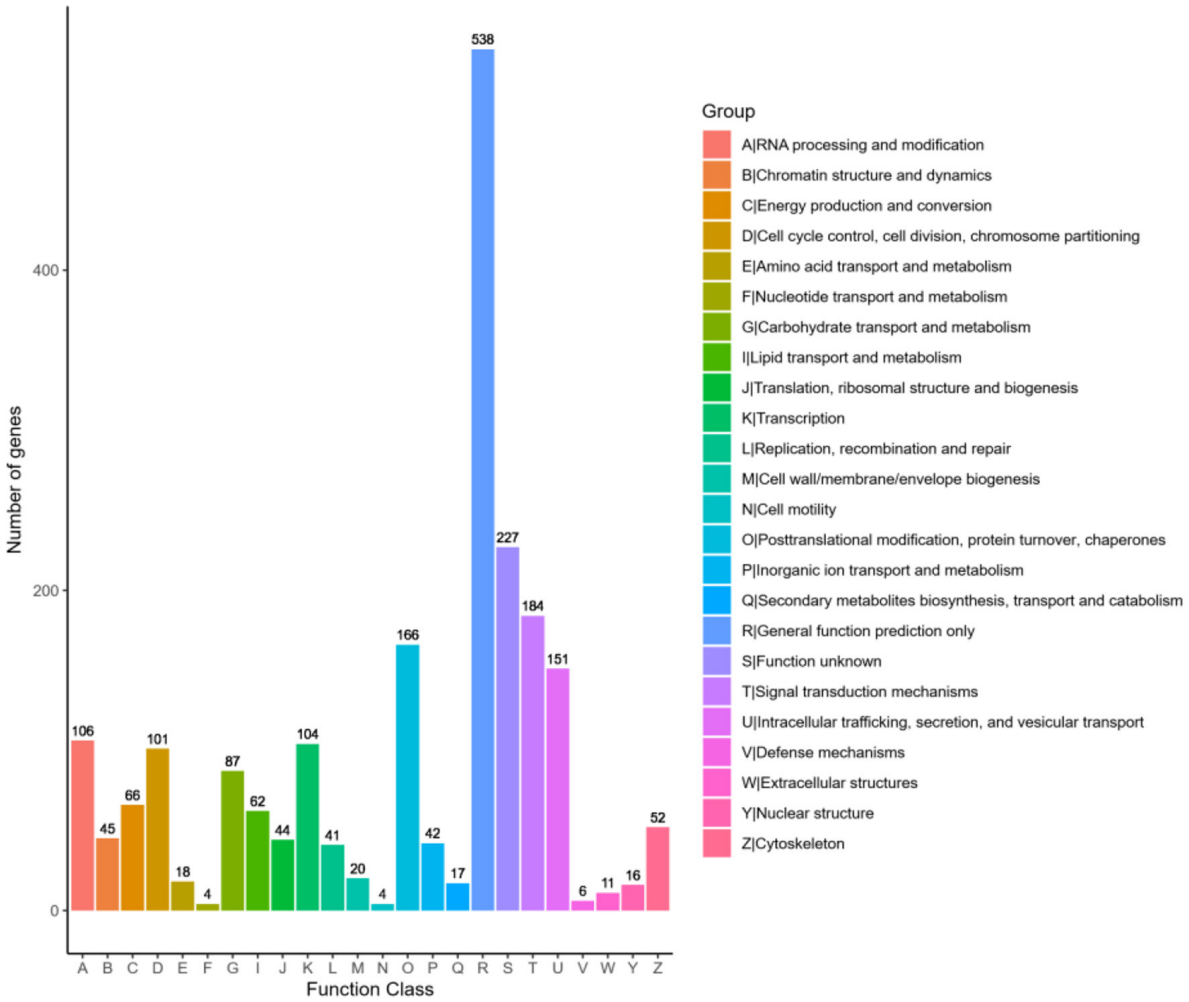
Statistical chart of KOG function annotation classification. The horizontal axis represents the various categories of KOG, while the vertical axis represents the number of genes. The proportion of genes in different functional categories reflects the metabolic or physiological trends relevant to the corresponding time period and environment.

The genes annotated by KOG are classified according to the KOG group. As shown in Figure 7, 1,871 KMR13 genes are annotated into 24 corresponding functions, with the largest number of function categories being general function predictions only. Secondly, the functional categories involving proteins are signal transduction mechanisms, post-translational modification, protein folding and protein turnover, intracellular trafficking, secretion and vesicular transport, and RNA processing and modification, though some additional functions are unknown. The COG annotation results revealed a large number of proteins with unknown functions in this strain, which are in need of further investigation.

#### 3.6.4 KEGG annotation

**Figure 8:**
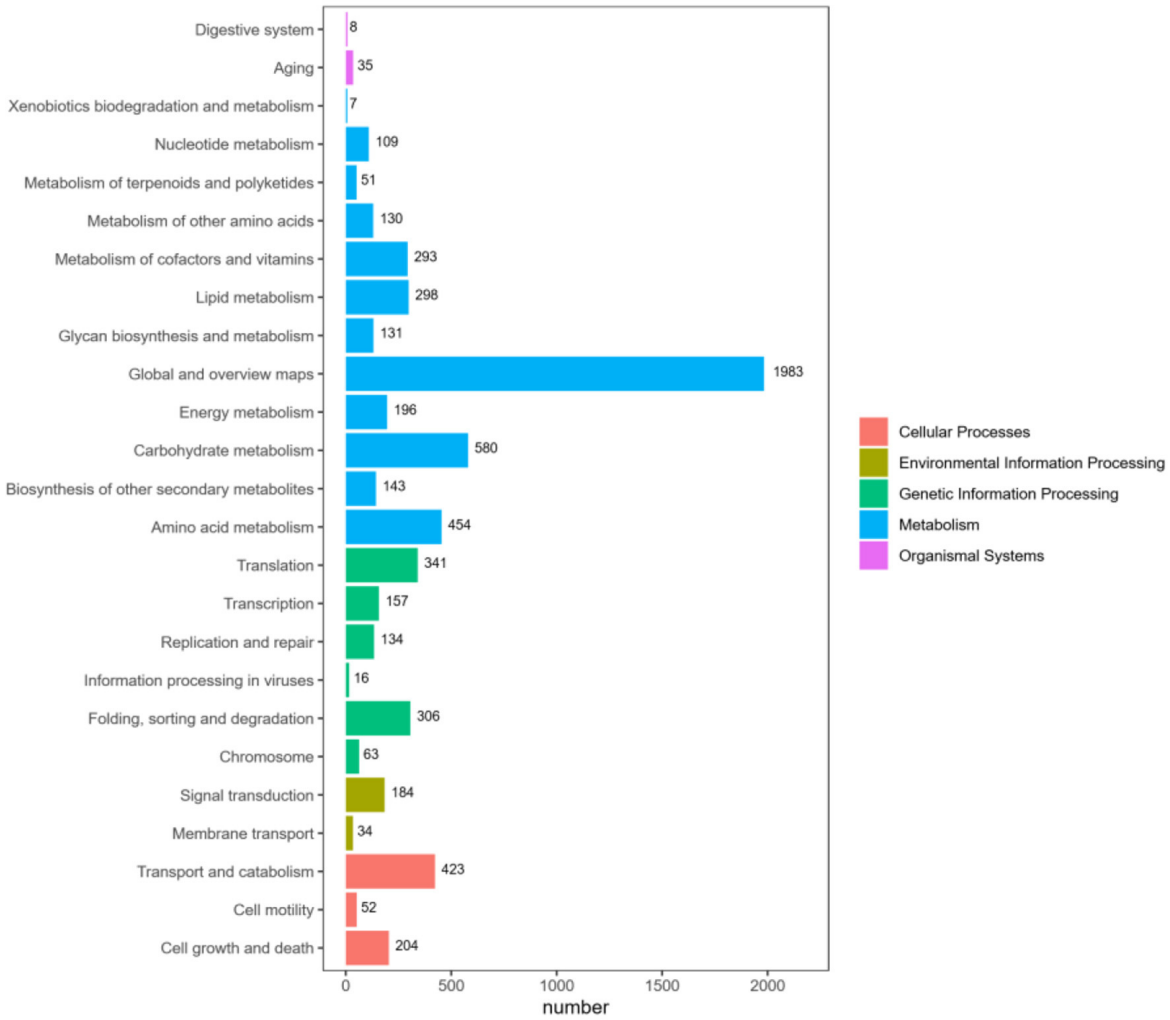
KEGG pathway functional classification map. The horizontal axis represents the number of genes annotated under each pathway category, while the vertical axis represents the pathway classifications. Different colors indicate the various major classifications to which they belong.

KEGG is an important database for systematic analysis of gene function and genome information. According to the KEGG metabolic pathway they participate in, 7,657 genes are annotated, and the results are shown in Figure 8. According to the global and overview maps (1,983 genes), carbohydrate metabolism (580 genes) is the most prevalent function, and amino acid metabolism (454 genes) is the second most prevalent. Numerous pathways involve a large number of proteins, including translation (341 genes), folding, classification, and degradation (306 genes) in genetic information processing; signal transduction in environmental information processing (184 genes); transport and catabolism (423 genes) in cellular processes; and cell growth and death (204 genes). The KEGG annotation results show that this strain is rich in carbohydrate metabolism.

#### 3.6.5 Pfam structural domain classification

**Figure 9:**
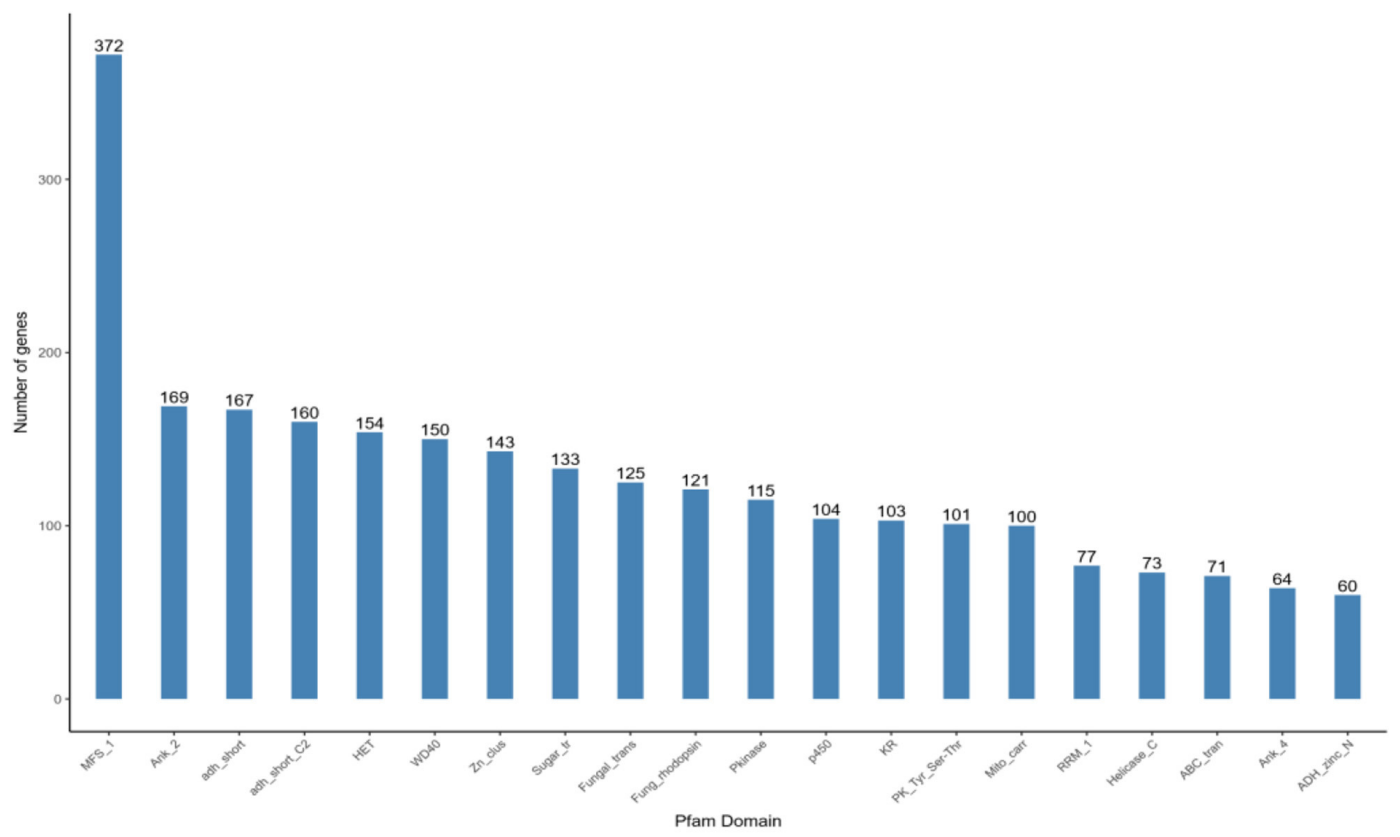
Pfam functional annotation categorization statistical chart. In the figure, the horizontal axis represents the names of protein families, while the vertical axis represents the number of genes aligned to each protein family.

A total of 7,911 genes were annotated based on the Pfam structural domain annotation, representing 63.06% of the total number of predicted genes. The genes annotated in each structural domain were subjected to statistical analysis, and the top 20 structural domains with the largest number of annotations were mapped and displayed. The results are presented in Figure 9, which depicts372 major facilitator transporter superfamilies (MFS_1), 169 Ankyrin_2(Ank_2), and 167 short-chain dehydrogenases (adh_short). Furthermore, 150 WD40 structural domains are involved in signal transduction and regulate the process of fungal cell differentiation.

### 3.7 Carbohydrate enzyme annotation

In the fungal strain KMR13, there are 600 genes were annated in CAZyme database (Table 6). Among these, the gene of 268 GHs exhibited the largest quantity, secondly, there are 151 AAs,88 GTs,53 CEs,25 PLs,15 CBMs. Among these, the GHs family and AAs family exhibited the largest numbers, account for 44.67% and 25.17% in overall families. Among these gene families, GH18 and others contain related genes encoding chitinases. From this, it was determined that strain KMR13 has the potential to degrade substances such as chitin.

**Table 6:** CAZyme annotation statistics in the KMR13 genome.

**Category**	**Number of genes**	**Percentage of total genes (%)**
GHs	268	44.67
GTs	88	14.67
PLs	25	4.17
CEs	53	8.83
AAs	151	25.17
CBMs	15	2.5

### 3.8 Chitinase gene expression analysis

**Figure 10:**
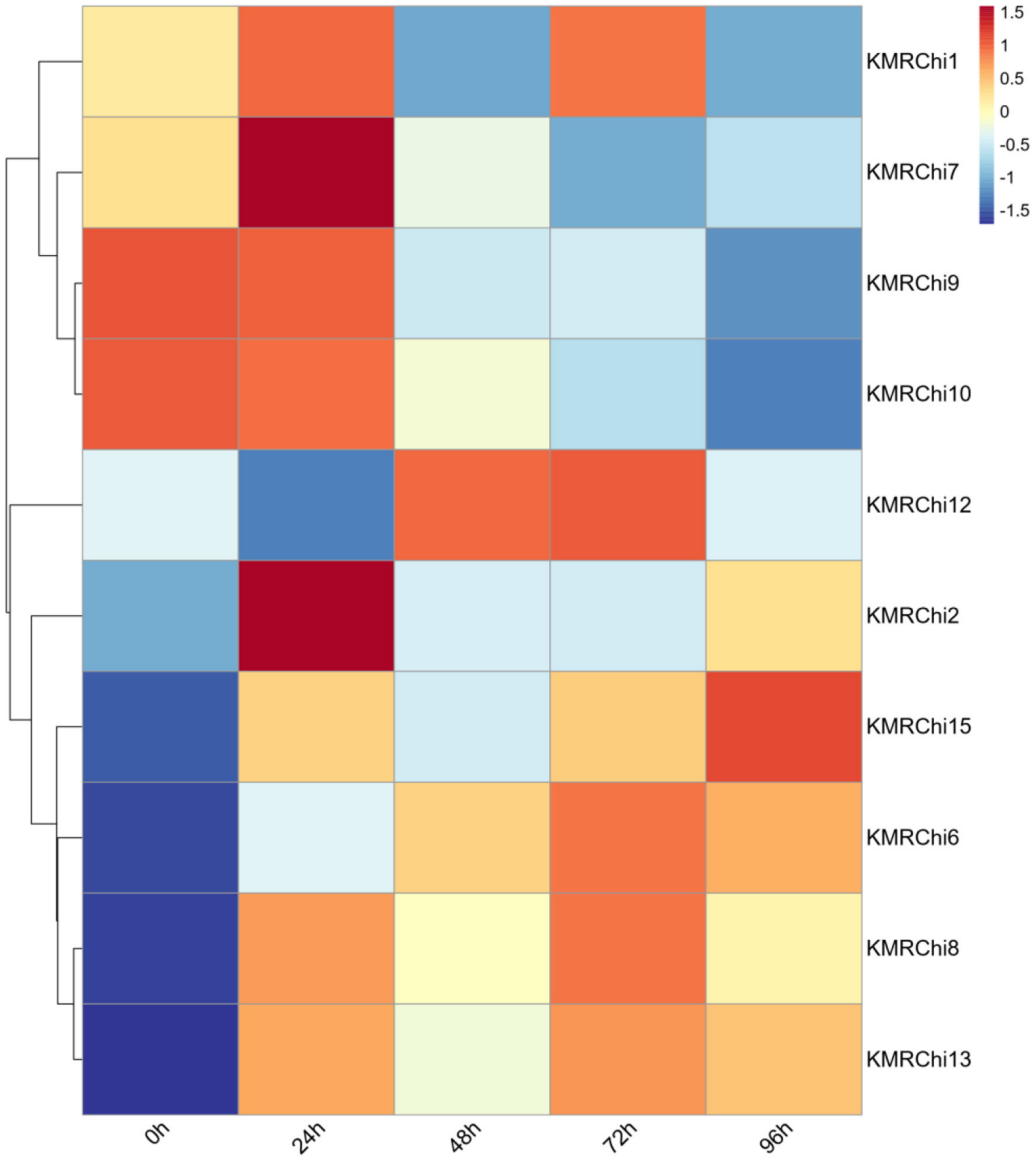
Expression of the GH18 gene family in strain KMR13 under spore induction.

A total of 15 GH18 family chitinase genes in strain KMR13 were screened, ordered and named according to the location of the genes on the chromosome (*KMRChi1-KMRChi15*). A total of 10 of the 15 chitinase genes in strain KMR13 were detected to be expressed in the transcriptome, of which *KMRChi3, KMRChi4, KMRChi5, KMRChi11*, and *KMRChi14* were not detected to be expressed; *KMRChi9* had the highest FPKM of 123.22 after 24 h of induction, followed by *KMRChi12*, which reached a peak of 81.99 after 72 h of induction. As seen in Figure 10, after spore wall induction, 10 chitinase genes showed expression differences between 0- 96 h of induction, of which a total of 5 genes, *KMRChi2, KMRChi6, KMRChi8, KMRChi13*, and *KMRChi15* were up-regulated in 0–96 h. These genes may play a possible role in the infestation of *P. pannosa KMRChi9* and *KMRChi10* were down-regulated in 0–96 h, which might be induced by the change of carbon source under induction, and *KMRChi12* was up-regulated in 48–72 h, which indicated that it might be suppressed in the early stage and functioned in the late stage of infestation.

**Figure 11:**
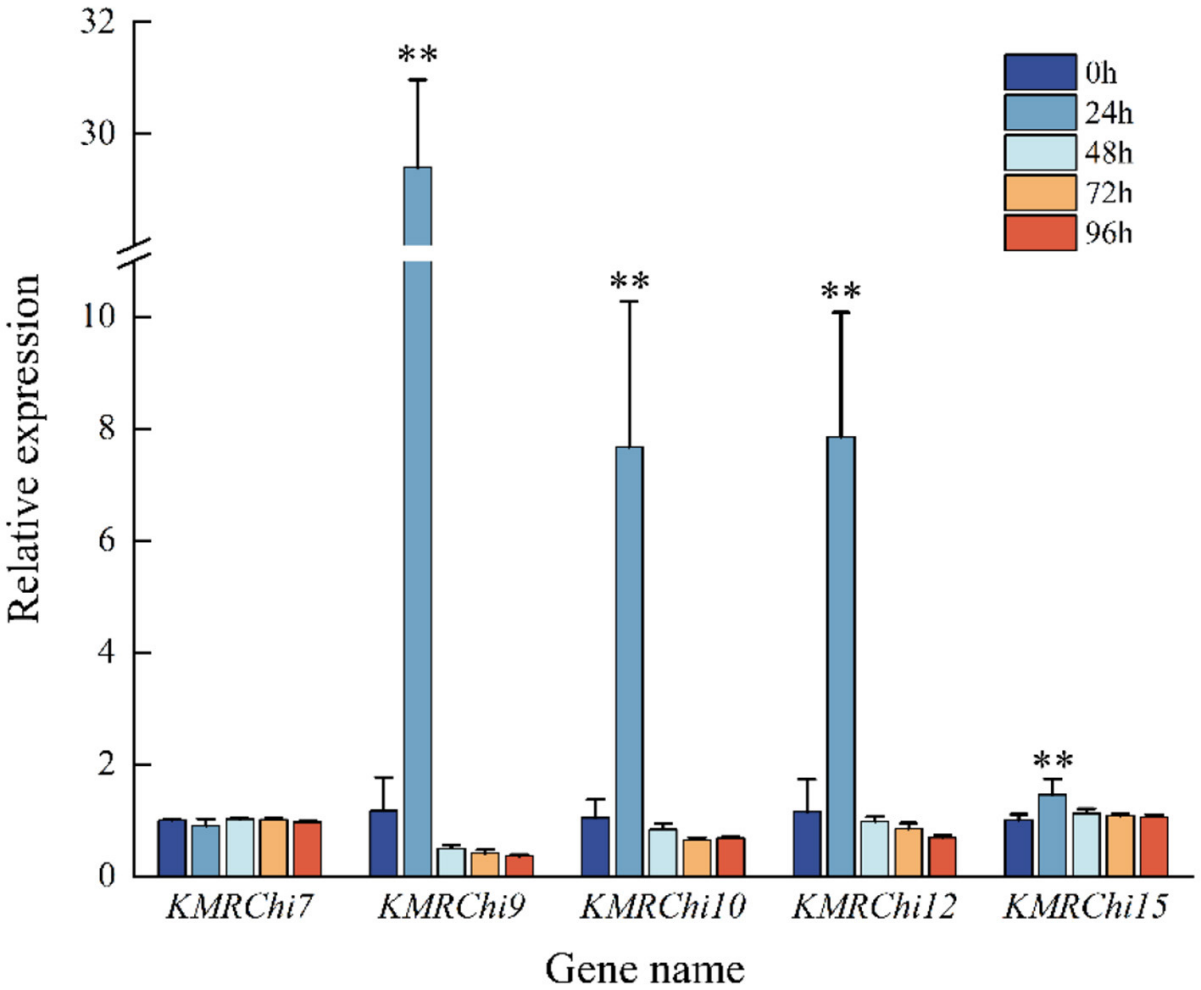
Relative expression of *KMRChis* under spore wall induction. Internal reference gene: actin; *n* = 3; *0.01 < *P* ≤ 0.05; **P ≤ 0.01.

Five genes with high expression or large fold difference in expression were screened for RT-qPCR validation, and the expression trends of the five *KMRChis* differed somewhat from the transcriptome results. From Figure 11 it can be seen that almost all of the chitinase genes were significantly up-regulated in expression at 24 h after the addition of spore induction, with the highest expression at 24 h, which showed a trend of induced expression. Five *KMRChis* showed significant up-regulation of expression at 24 h after induction, among which *KMRChi9* and *KMRChi10* were up-regulated up to 25.09-fold and 7.3-fold, respectively.

## 4 Discussion

Based on the data pertaining to the KMR13 genome assembly size, N50, second- generation alignment rate and coverage, and the proportion of Complete BUSCOs (C), the KMR13 genome appears to have good continuity, consistency and integrity, and its data are rigorous with strong reference value. Mycoparasitism is a common phenomenon in plant pathogenic fungi in nature, in biological control with mycoparasitic fungi made of live cell preparation is different from chemical agents, non-toxic and harmless, will not have harmful side effects on the target plant, will not affect the environment, the control effect has a long-lasting, it is the mainstream direction of biological control (Qiao and Zong, 2002), more and more by the national plant pathologists, research on the mechanism of mycoparasitism by means of genome sequencing has greatly facilitated the application of mycoparasitic fungi in biological control.

Fungal cell walls are mainly composed of polysaccharides (80%) and proteins (3%−20%), with lesser amounts of lipids, pigments and inorganic salts. Filamentous fungal cell walls are made up of dextran, glycoproteins, proteins and chitin from the outside to the inside, with β-glucan, chitin (or chitosan in some fungi) and cellulose microfibrils constituting a scaffolding that is responsible for the strength and shape of the cell wall (Steyaert et al., 2003). Cell wall is a necessary condition for the survival of fungal cells. Cell wall degradation will affect the osmotic pressure balance of fungal cells; that is, excessive internal swelling pressure will eventually lead to cell degradation. Therefore, disrupting cell wall synthesis is a potential antifungal mechanism. Chitinase genes were mined from the KMR13 genome and screened for mycoparasitism-associated chitinase genes by detecting the expression of genes at different time periods of spore induction, and it was found that the up-regulated expression of *KMRChis* after induction is likely to play a role in destroying the spore wall in the mycoparasitism process of strain KMR13. This strain may catalyze the degradation of chitin in the cell wall of phytopathogenic fungi by secreting cell wall degrading enzymes such as chitinase, which results in inactivation of the cell wall lysis and spillage of inclusions, which may be one of the mechanisms of mycoparasitism of strain KMR13. *T. brevicompactum* DTN19, isolated from the pathogenic rootstocks of saffron, exerted a good inhibitory effect on the growth and development of *Fusarium oxysporum* and contained a large number of genes encoding proteins related to cellulase, chitinase, β-glucosidase, iron carrier, nitrogen cycling, and sulfate transport proteins, as analyzed by genome sequencing (Tian et al., 2024). This study also confirmed that strain KMR13 has a gene encoding chitinase. The mycoparasitism of strain KMR13 is similar to that of *Trichoderma*, which may be due to the secretion of spore wall degrading enzymes to hydrolyze the host cell wall, thus absorbing the host cytoplasm as a nutrient component for parasitic growth. The microscopic observation of strain KMR13 mycoparasitism assay was consistent with the above, with the spore cell wall fragmentation and the release of inclusions, and it can be speculated that its main myoparasitism mechanism may also be for the secretion of cell wall degrading enzymes.

The expression levels of many genes encoding fungal cell wall degrading enzymes are significantly regulated during plant pathogenic fungal infestation, and different species of fungal cell wall degrading enzymes are involved in this biological process and in different proportions at different phases of infection, suggesting that the process of reorganization or modification of the fungal cell wall is subject to a complex and stringent regulation (Lyu et al., 2015). In this study, we found that the expression of some chitinase genes in fungal cell wall degrading enzymes was down-regulated after spore induction, and it was speculated that the reason for this might be affected by time and nutrient source on the one hand, and the study showed that the expression of CAZymes is complex, and there are different regulatory mechanisms at different developmental stages of fungal growth, which are affected by light and temperature in addition to carbon and nitrogen sources (Donzelli and Harman, 2001); On the other hand, cell wall degrading enzymes are not only involved in fungal mycoparasitism or digestion and breakdown of chitin, but the same proteins involved in fungal mycoparasitism during autolysis and starvation of aging fungi are also mainly responsible for the breakdown of exogenous chitin, the recycling of chitin in the fungi's own cell wall and the remodeling of the fungal cell wall during growth (Hu and Leger, 2004; Gruber and Seidl-Seiboth, 2012). MAP kinase cascade reaction (Zeng et al., 2012), NADPH oxidase (Wei et al., 2016), oxalate decarboxylase (Zeng et al., 2014), transcription factor (Gao et al., 2016), peroxisome, and heat shock factor have been proven to participate in the process of mycoparasitism (Hamid et al., 2013). In addition, the transcription of genes encoding cell wall degrading enzymes is also related to the regulation of transcription factors. In the study of *Trichoderma reesei* degrading cellulose and hemicellulose, it is shown that transcription activating factors such as XYR1 and ACE2, as well as CRE1 and ACE1 as inhibitory factors, participate in the expression of intracellular enzymes (Akimitsu et al., 2004; Seiboth et al., 2012). Because the mycoparasitism of strain KMR13 is similar to *Trichoderma*, transcription factors may also participate in the mycoparasitism of KMR13, which needs to be verified later.

Field greenhouse tests showed that a suspension of conidia of strain KMR13 was less effective in controlling Chinese rose powdery mildew caused by *P. pannosa* than under indoor conditions. Carroll and Wilcox (2003). found that the incidence and severity of powdery mildew disease increased with increasing relative humidity, so the present study may be attributed to the increased probability of infestation by the pathogenic fungi due to low nighttime temperatures and high relative humidity in field greenhouses, whereas indoor environments, due to their more stable temperatures and relative humidity, do not have the potential for high nighttime relative humidity, resulting in less effective control in field greenhouses than in indoor conditions. Relative humidity should be accurately controlled in subsequent studies of control effects. Biocontrol agents have a good background of action against host pathogens, but their use and effectiveness in this area is limited. The lack of field application reliability of biological control agents and their failure to provide consistent disease control under field conditions potentially contribute to their low dissemination on the market (Spadaro and Droby, 2016; Janisiewicz and Jeffers, 1997). In addition to the above mentioned problems, the workflow from the discovery of bioactive compounds to the effective preparation of final products is still quite complicated; in most cases, it is a long iterative process involving several steps. Therefore, it is necessary to improve the regulatory barriers and workflow-related procedures to overcome the challenges of the biocontrol agent market. The adoption and widespread use of biocontrol agents will make it possible to produce more natural, healthier, and safer foods with no or low chemical residue values (Abbey et al., 2019; Belinato et al., 2018).

## 5 Conclusion

KMR13 disrupted the cell wall of *P. pannosa* spores, inducing leakage of intracellular content while showing no phytotoxicity to host plants. Conidial suspension of strain KMR13 had lower field control efficacy against Chinese rose powdery mildew compared to its greenhouse pot control efficacy, but it still y suppressed Chinese rose powdery mildew caused by *P. pannosa*, supporting its potential application in agricultural biological control. In this study, we reported the whole genome sequence information of strain KMR13 for the first time and analyzed its gene functional annotation, predicted that the genome of strain KMR13 contains relevant chitinase genes for degrading the cell wall of pathogenic fungi, and combined with the transcriptome and RT-qPCR data, found that the up-regulated expression of *KMRChis* induced is likely to play the role of destroying the spore wall in the process of mycoparasitism of strain KMR13. Therefore, it is speculated that strain KMR13 may catalyze the degradation of chitin in the cell wall of phytopathogenic fungi through the secretion of chitinase, thus causing the cell wall to be inactive and the inclusions to overflow to achieve the effect of plant disease control. This provides a theoretical basis for a better understanding of the exploitation of strain KMR13 for biocontrol and the elucidation of the mycoparasitic mechanism.

## Data Availability

The sequencing data for this project are available via the National Center for Biotechnology Information (NCBI) repository under BioProject: PRJNA1186384; BioSample: SAMN44754513; SRA: SRR31378760.
